# Immune-Related Myocarditis Presenting as Upper Abdominal Discomfort in a Patient With Metastatic Subungual Melanoma Treated With Nivolumab

**DOI:** 10.7759/cureus.93683

**Published:** 2025-10-01

**Authors:** Rana Tokioka, Natsuko Saito-Sasaki, Yoko Amakata, Yu Sawada

**Affiliations:** 1 Dermatology, University of Occupational and Environmental Health, Kitakyushu, JPN

**Keywords:** case report, ecg, irae, myocarditis, nivolumab

## Abstract

Immune checkpoint inhibitors (ICIs) have transformed the treatment of advanced melanoma, but they are associated with immune-related adverse events, including myocarditis, which, although rare, can be life-threatening. We report a case of suspected nivolumab-induced myocarditis in a 76-year-old woman with metastatic subungual melanoma. After receiving two doses of nivolumab, she experienced mild upper abdominal discomfort without chest pain or dyspnea. Laboratory tests revealed elevated creatine kinase and troponin T levels, and subsequent ECG showed new negative T waves. Although cardiac MRI and biopsy were not performed, the diagnosis was clinically suspected based on biomarker elevation, ECG changes, and the exclusion of coronary artery disease. High-dose corticosteroid therapy was initiated, leading to normalization of biomarkers and resolution of ECG abnormalities. The patient recovered without recurrence. This case emphasizes the importance of early recognition and treatment of ICI-related myocarditis, even in patients with rare melanoma subtypes and atypical presentations.

## Introduction

Immune checkpoint inhibitors (ICIs), including programmed cell death-1 (PD-1) inhibitors such as nivolumab, have revolutionized cancer treatment and brought survival benefits to patients with advanced melanoma. However, they are also associated with a distinct spectrum of immune-related adverse events (irAEs), and among these, myocarditis is rare but stands out as one of the most immediately life-threatening. Immune checkpoints such as PD-1 and cytotoxic T-lymphocyte-associated antigen-4 (CTLA-4) are inhibitory pathways that physiologically function to maintain self-tolerance and prevent excessive immune activation. Tumor cells frequently exploit these pathways to escape immune surveillance by expressing ligands such as PD-L1. ICIs block these inhibitory signals, thereby restoring cytotoxic T-cell activity against tumors and producing potent antitumor effects. At the same time, this loss of immune tolerance underlies the development of irAEs. While blockade of these pathways enhances anti-tumor immunity, it simultaneously disrupts peripheral tolerance and permits autoreactive T-cell activation, which underlies the development of irAEs. Though the incidence is estimated between 0.09% and 1.14% [1,2], mortality can reach up to 50% [3]. This underscores its catastrophic potential and highlights the importance of timely recognition. Myocarditis often develops within the first month of ICI therapy, and over 80% of cases arise within three months [4]. Diagnosis is often difficult due to the nonspecific symptoms and early imaging findings. Given the rapid progression and potentially fatal outcome, early clinical suspicion and prompt corticosteroid intervention are critical.

Here, we present a case of suspected nivolumab-induced myocarditis in a patient with metastatic subungual melanoma. This case highlights the importance of early recognition and treatment, even in the absence of definitive diagnostic criteria.

## Case presentation

**Figure 1:**
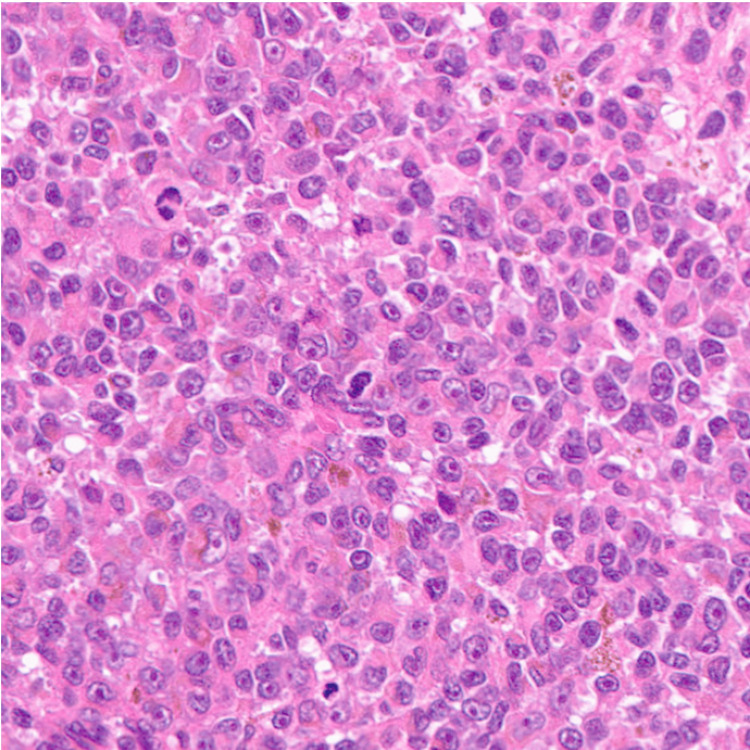
Histological examination Histopathology of the primary tumor. Hematoxylin and eosin staining of the primary subungual melanoma showing atypical melanocytic proliferation within the dermis.

**Figure 2:**
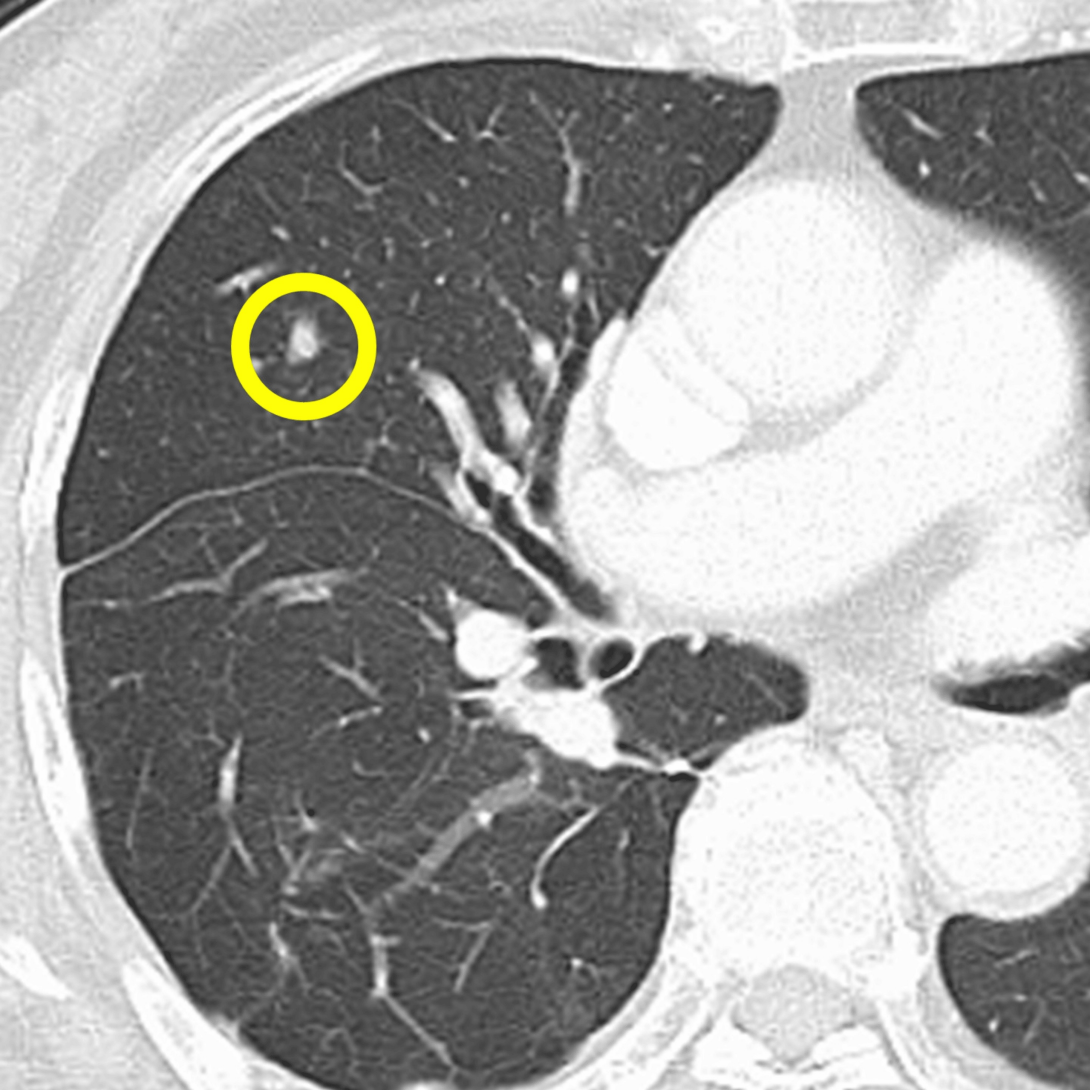
CT imaging Contrast-enhanced CT image showing metastatic lesions in the lung (the yellow circle).

A 76-year-old woman with a history of subungual malignant melanoma (pT3aN0M0, stage IIA, BRAF wild type) underwent resection of the primary tumor (Figure 1). Two years later, she developed metastases in the left inguinal and external iliac lymph nodes, as well as the lungs (Figure 2). Following left inguinal lymph node dissection, she underwent intensity-modulated radiation therapy (IMRT, 60 Gy) and was started on nivolumab 240 mg on day 13. The second dose was given on day 27 without immediate adverse events. On day 31, she noted mild upper abdominal discomfort without chest pain, palpitations, or dyspnea. This resolved spontaneously. Although the discomfort was vague and localized to the upper abdomen, given the patient’s ongoing ICI therapy and the known potential for atypical presentations of immune-related myocarditis, this symptom prompted increased clinical vigilance.

On day 34, routine blood tests revealed an elevated creatine kinase (CK, 580 U/L), and subsequent examination showed elevated troponin T (0.062 ng/mL). There were no concurrent ECG or echocardiographic abnormalities at that time. Given the elevation of CK, the possibility of immune-related myositis was also considered. However, the patient had no muscle weakness, myalgia, or ocular symptoms, and there were no physical or imaging findings suggestive of myositis. At that point, we also considered the possibility of early-stage myocarditis, including viral myocarditis, and consulted the cardiology department for further evaluation. Because the patient was clinically stable and the initial symptoms were atypical, myocarditis was not initially assumed, and the cardiology team hesitated to pursue cardiac MRI or endomyocardial biopsy. Nevertheless, myocarditis remained the leading clinical concern in the differential diagnosis. Because of the biomarker elevation and recent ICI exposure, we emphasized the need for close clinical monitoring and advised the patient of the potential for clinical deterioration. Frequent follow-up assessments were arranged to ensure any emerging signs of myocarditis could be identified and addressed promptly.

**Figure 3:**
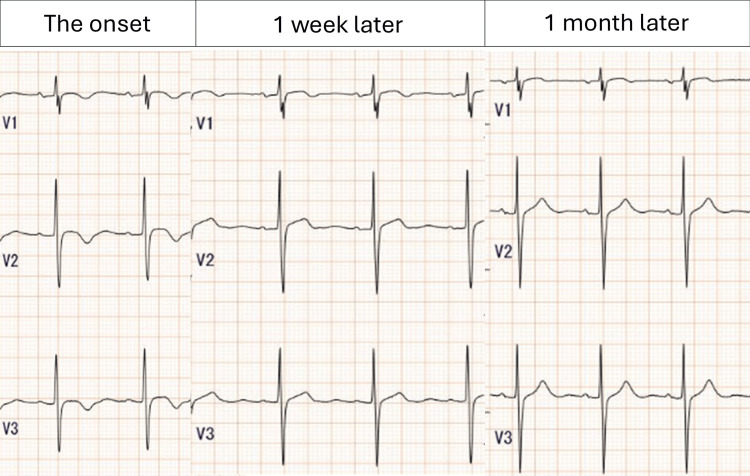
Electrocardiographic findings Serial electrocardiograms. Electrocardiograms obtained at onset (left), one week later (middle), and one month later (right). At onset, newly developed negative T waves were observed in leads V1-V3, consistent with early myocarditis. These abnormalities partially persisted at one week but had completely resolved by one month following corticosteroid therapy.

On day 37, a repeat ECG revealed newly developed negative T waves in leads V1-V3 (Figure 3). Although the patient remained hemodynamically stable and asymptomatic, these changes strengthened the suspicion of early immune-related myocarditis. Cardiac MRI and biopsy were not performed. Thus, the diagnosis was based on biomarker elevation, evolving ECG changes, exclusion of coronary disease on CT angiography, and subsequent clinical improvement with corticosteroid therapy. We fully acknowledge that a high index of suspicion alone is not sufficient to establish the diagnosis. Confirmatory investigations, such as cardiac MRI or endomyocardial biopsy, are essential not only for diagnostic certainty but also for prognostication, and their omission represents a limitation of this case. With the patient’s informed consent after thorough explanation of the potential for rapid progression, high-dose intravenous methylprednisolone (1,000 mg/day) was administered for three days, followed by oral prednisolone at 1 mg/kg/day. After initiating corticosteroid therapy, CK and troponin levels gradually normalized, ECG changes resolved (Figure 3), and no further symptoms occurred. Serial measurements of cardiac biomarkers showed that troponin T rose to a peak of 0.896 ng/mL and then progressively declined to 0.012 ng/mL, in parallel with the resolution of ECG changes and clinical improvement. These serial changes in cardiac biomarkers, together with the evolving ECG findings and clinical course, strongly supported the diagnosis of ICI-related myocarditis. The corticosteroids were tapered, and she was discharged home on day 78 without recurrence. The patient has been followed monthly since discharge, and no recurrence of myocarditis or other irAEs has been observed to date. According to CTCAE, the suspected immune-related myocarditis was classified as Grade 3.

## Discussion

This case presents several clinically relevant and novel features. First, the initial manifestation was mild and atypical, consisting only of upper abdominal discomfort. In this case, the abdominal discomfort may be explained by two plausible physiological mechanisms: [1] diaphragmatic pericardial inflammation stimulating the phrenic nerve, resulting in referred pain to the upper abdomen [5]; and [2] activation of vagal afferents by cardiac inflammation, consistent with the Bezold-Jarisch reflex, leading to nausea and epigastric discomfort [6,7]. At least one published case of ICI-related myocarditis has described initial presentation with abdominal pain during treatment [8]. These atypical manifestations may obscure the diagnosis, underscoring the importance of including myocarditis in the differential diagnosis even in the absence of classical cardiac symptoms.

Second, this is the first reported case of suspected ICI-related myocarditis in a patient with subungual melanoma. While ICI-associated myocarditis has been reported in cutaneous and mucosal melanoma, reports involving subungual melanoma as an uncommon subtype of acral melanoma are lacking. Importantly, current evidence does not suggest that tumor origin plays a role in the pathogenesis of ICI-related myocarditis. Subungual melanoma is typically thought to have a lower tumor mutational burden (TMB) and reduced immunogenicity compared to sun-exposed melanoma based on the acral melanoma subtypes [9,10]. Thus, this case highlights that ICI-related myocarditis can occur even in rare tumor types, emphasizing the need for clinical vigilance regardless of tumor subtype.

Third, the diagnosis of myocarditis was suspected based on clinical judgment and treated promptly, despite the absence of definitive diagnostic criteria. As previous studies have shown, delayed recognition of ICI-associated myocarditis is associated with poor prognosis [4]. In this case, early administration of corticosteroids led to symptom resolution, suggesting that timely clinical decision-making can be critical in preventing disease progression, even when the presentation is subtle and the diagnosis is uncertain.

We fully acknowledge that a high index of suspicion alone is not sufficient to establish the diagnosis. Confirmatory investigations, such as cardiac MRI or endomyocardial biopsy, are essential not only for diagnostic certainty but also for prognostication, and their omission represents a limitation of this case. This case underscores the diagnostic difficulties of ICI-related myocarditis, particularly presenting with atypical, non-cardiac symptoms such as vague abdominal discomfort and isolated CK elevation. It highlights the importance of considering myocarditis even in the absence of chest pain or dyspnea and demonstrates the diagnostic value of close monitoring with serial ECGs to capture evolving changes. These clinical nuances provide educational value for physicians managing patients on ICI therapy.

As with any single case report, the findings cannot be generalized to the broader patient population. This report highlights atypical presentations of ICI-related myocarditis and raises the hypothesis of increased risk in patients with subungual melanoma; however, these observations require validation in larger cohorts or registries.

## Conclusions

Taken together, this case broadens the recognized clinical spectrum of ICI-associated myocarditis. It underscores the importance of considering myocarditis in patients presenting with atypical symptoms, even in the absence of conventional risk factors or classical tumor types. Prompt recognition and intervention based on clinical suspicion may be key to favorable outcomes in such cases.
